# Down-Regulation of AP-4 Inhibits Proliferation, Induces Cell Cycle Arrest and Promotes Apoptosis in Human Gastric Cancer Cells

**DOI:** 10.1371/journal.pone.0037096

**Published:** 2012-05-16

**Authors:** Xinghua Liu, Bo Zhang, Yan Guo, Qi Liang, Changyao Wu, Lei Wu, Kaixiong Tao, Guobin Wang, Jianying Chen

**Affiliations:** 1 Department of General Surgery, Union Hospital, Tongji Medical College, Huazhong University of Science and Technology, Wuhan, China; 2 Department of Maternal and Child Health, School of Public Health, Tongji Medical College, Huazhong University of Science and Technology, Wuhan, China; University of Missouri-Columbia, United States of America

## Abstract

**Background:**

AP-4 belongs to the basic helix-loop-helix leucine-zipper subgroup; it controls target gene expression, regulates growth, development and cell apoptosis and has been implicated in tumorigenesis. Our previous studies indicated that AP-4 was frequently overexpressed in gastric cancers and may be associated with the poor prognosis. The purpose of this study is to examine whether silencing of AP-4 can alter biological characteristics of gastric cancer cells.

**Methods:**

Two specific siRNAs targeting AP-4 were designed, synthesized, and transfected into gastric cancer cell lines and human normal mucosa cells. AP-4 expression was measured with real-time quantitative PCR and Western blot. Cell proliferation and chemo-sensitivity were detected by CCK-8 assay. Cell cycle assay and apoptosis assay were performed by flow cytometer, and relative expression of cell cycle regulators were detected by real-time quantitative PCR and Western blot, expression of the factors involved in the apoptosis pathway were examined in mRNA and protein level.

**Results:**

The expression of AP-4 was silenced by the siRNAs transfection and the effects of AP-4 knockdown lasted 24 to 96 hrs. The siRNA-mediated silencing of AP-4 suppressed the cellular proliferation, induced apoptosis and sensitized cancer cells to anticancer drugs. In addition, the expression level of p21, p53 and Caspase-9 were increased when AP-4 was knockdown, but the expression of cyclin D1, Bcl-2 and Bcl-x_L_ was inhibited. It didn't induce cell cycle arrest when AP-4 was knockdown in p53 defect gastric cancer cell line Kato-III.

**Conclusions:**

These results illustrated that gene silencing of AP-4 can efficiently inhibited cell proliferation, triggered apoptosis and sensitized cancer cells to anticancer drugs in vitro, suggesting that AP-4 siRNAs mediated silencing has a potential value in the treatment of human gastric cancer.

## Introduction

Although the incidence and mortality rates associated with gastric cancer have gradually decreased in recent years in most areas of the world [1], [2], gastric cancer remains a worldwide health burden, and remains the most common cause of cancer related deaths with little improvement of long-term survival. The most efficient treatment of gastric cancer was completely surgical removal of the neoplastic tissue with D2 lymphadenectomy. In addition adjuvant chemotherapy and radiotherapy have assisted to improve prognosis. Even so, the 5-year survival remained very poor [1], [3]. More than one million new cases were diagnosed each year, especially in East Asia, like Japan, Korea, and China. In these countries, gastric cancer remains the most common cause of cancer related deaths, and the precise pathogenesis remains unknown [3].

Transcription factors are important regulatory components [4]. They belonged to the helix-loop-helix family and played an important role in cell proliferation and differentiation, cell lineage determination, expression of intracellular genetic information, and other essential processes [5]. As a member of the basic helix-loop-helix leucine-zipper (bHLH-LZ) subgroup of bHLH proteins [6], activating enhancer binding protein 4(AP-4) was initially identified as a cellular protein that bound to the simian virus 40 (SV40) enhancer and activated the viral late gene transcription [7]. AP-4 is a ubiquitously expressed transcription factor and may control transcriptional networks during cellular differentiation by forming homodimers and binding to the symmetrical DNA sequence, CAGCTG [7], [8], [9], [10], [11], [12]. AP-4 is a ligand for immunoglobulin-kappa promoter E-box elements, which may be implicated to immunodeficiency diseases [13], [14]. In addition, unlike other HLH proteins, AP-4 contains two additional protein dimerization motifs consisting of leucine repeat elements LR1 and LR2. Hence, AP-4 presents a specific tripartite dimerization structure, suggesting that AP-4 may interact with a wide variety of transcription factors [8], [14]. AP-4 regulates the expression of some genes [9], [15], [16], [17], [18], [19], [20], [21]. For instance, it activates the transcription of hMTIIA gene and participates in the regulation of human proenkephalin expression [22], and it may play a role in the expression of the pancreatic exocrine gene family [23]. Recently, the overexpression of AP-4 was reported in the colorectal cancer, breast cancer and prostate cancer [9], [18], [24].

RNA interference (RNAi) is a process of sequence specific post-transcriptional gene silencing initiated by double-stranded RNA [25], which can lead to the silencing of specific cellular gene and provide a powerful reverse genetics approach to analyze gene functions both in vitro and in vivo [26]. Currently the most widely used nucleic acid-based sequence-specific gene silencing molecules were small interfering RNAs [27], named siRNAs, which consists of symmetrical duplexes of 19–21 base pairs [28]. The siRNA method could inhibit target gene expression with specificity, efficiency and endurance [29].

We reported previously that AP-4 was overexpressed in gastric cancer and that it may be associated with the poor prognosis [30]. In the present study, we further examined the AP-4 function in human gastric cancer cell with RNA interference.

## Results

### Specific siRNA targeting the AP-4 expression in human gastric cancer cells and human normal mucosa cells

**Figure 1:**
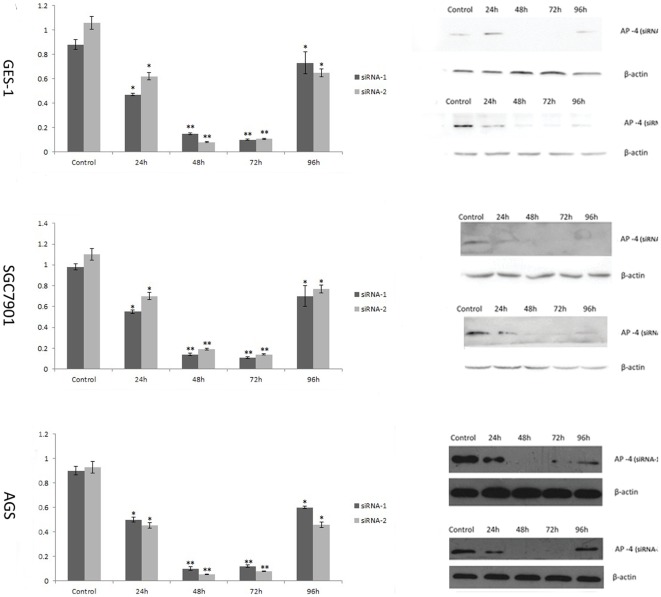
AP-4 specific-siRNAs suppressed the AP-4 expression in gastric cancer cells and human normal mucosa cells. The different siRNAs were transfected into the cells for 24 h, 48 h, 72 h, and 96 h. The mRNA and protein expression were examined by real-time quantitative PCR and Western blot. AP-4 specific-siRNAs could effectively inhibit the gene expression. The induced suppression of AP-4 expression started at 24 hours, and the inhibition ratio decreased after 72 hours. The most efficient time point were 48 h and 72 h, the relative levels of mRNA transcripts significantly decreased by nearly 90%. There was statistical significance between AP-4 siRNAs groups and control groups. (* p<0.05; ** p<0.01).

To evaluate the effect of the siRNA-mediated silence of the AP-4 gene expression, the control siRNA and AP-4 specific siRNAs were transfected into the cells for 24 h, 48 h, 72 h, and 96 h. The efficacy in down-regulating expression of AP-4 gene was detected by real-time quantitative PCR and western blot. As shown in Figure 1, AP-4 specific siRNAs could effectively inhibit the gene transcription and translation. The mRNA and protein levels of AP-4 were decreased with AP-4 siRNA transfection group but not in the control siRNA (Figure 1). The siRNAs induced suppression of AP-4 expression could be detected in 24 hours after transfection. However, the inhibition ratio decreased 72 hours after transfection. The most efficient time point were between 48 h to 72 h in suppress expression of AP-4. The relative levels of mRNA transcripts significantly decreased by nearly 90% in siRNA-1 group, and 95% in siRNA-2 group. There was statistical significance between siRNAs group and the control group.

### Down-regulation of AP-4 expression inhibited the cell proliferation and sensitized human gastric cancer cells to anticancer drugs

**Figure 2:**
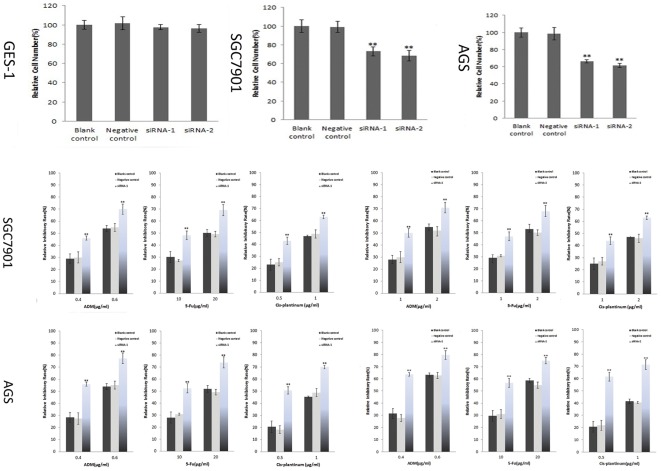
Down-regulation of AP-4 expression inhibits the proliferation of gastric cancer cells, and enhances the chemo-sensitivity. Forty-eight hours post-transfection, the cell proliferation and inhibitory effects of different concentration of 5-FU, ADR or Cis-plantinum were evaluated by CCK-8 assay. The results indicated that AP-4 specific-siRNAs could inhibit the proliferation of gastric cancer cells but not the normal mucosa cells (p<0.01) and enhance the chemo-sensitivities of gastric cancer cells to 5-FU, ADR or Cis-plantinum (p<0.0001). There was statistical significance between AP-4 siRNAs groups and control groups (* p<0.05; * p<0.01).

To examine the effect of AP-4 specific siRNAs on cell proliferation and chemo-sensitivity of the cancer cells, 20 nM of AP-4 specific siRNAs or control siRNA were transfected and the cell proliferation was determined by CCK-8 assay 48 hours after transfection. We found that knockdown AP-4 could inhibit the proliferation of gastric cancer cell lines SGC7901 and AGS (Figure 2), but not in normal mucosa cell line GES-1 when compared with the control siRNA transfection in 48 hours (Figure 2). These results indicated that AP-4 siRNAs attenuated the cell proliferation of gastric cancer cells in vitro. Furthermore, we investigated the role of AP-4 in the regulation of chemo-sensitivity of human gastric cancer cells. We compared the drug sensitivity of AP-4 siRNAs with that of control siRNA or mock cells and found that the relative inhibition rates of AP-4 specific siRNAs were significantly higher than that of control siRNA or mock cells (p<0.0001) (Figure 2). In addition, AP-4 siRNAs significantly enhanced the sensitivity of cells to ADR, 5-FU or cis-plantinum treatment at two differently doses (Figure 2), these suggested that down-regulation of AP-4 may have a beneficial effect in the sensitivity of chemotherapy.

### Silencing of the AP-4 expression induced cell cycle arrest and modulated the expression of p53, p21 and cyclin D1

**Figure 3:**
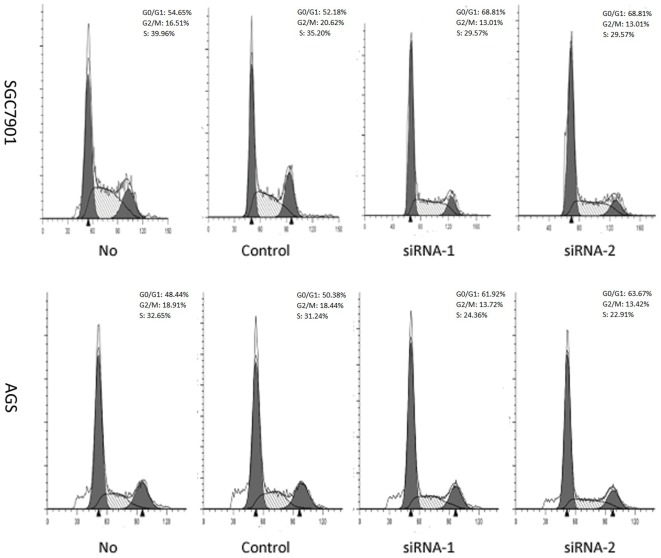
The effect of AP-4 on gastric cancer cell cycle was investigated by flow cytometry. Forty-eight hours post-transfection, the cells were harvested and stained with propidium iodide, and proportion of cells in each phase of cell cycling was assayed. In the graph, the proportion of cells at G0/G1 phases in the cell transfected with AP-4 siRNAs was significantly higher than that of the mock cells or control siRNA. (* p<0.05; ** p<0.01).

The effect of AP-4 on cell cycle progression was investigated. Human gastric cancer cells were transfected with 20 nM control siRNA, and the AP-4 specific siRNAs for 48 h, respectively, followed by propidium iodide staining and flow cytometry analysis of cell cycle. While cells transfected with control siRNA progressed through different phases of cell cycle, cells transfected with AP-4 specific siRNAs displayed significantly higher frequency of cells at the G0/G1 phases (SGC7901/siRNA-1: 68.81%; SGC7901/siRNA-2: 68.97%; AGS/siRNA-1: 61.92%; AGS/siRNA-2: 63.67%) and a lower frequency of cells at S-phase (SGC7901/siRNA-1: 29.57%; SGC7901/siRNA-2: 29.84%; AGS/siRNA-1: 24.36%; AGS/siRNA-2: 22.91%). The percentage of cells at G0/G1 phases in the cell transfected with AP-4 siRNAs was significantly higher than that of the mock cells (SGC7901: 54.65%; AGS: 48.44%) (p<0.05) or control siRNA (SGC7901/control siRNA: 52.18%; AGS/control siRNA: 50.38%) (Figure 3). Therefore, transfection with AP-4-specific siRNAs induced cell cyclin arrest at G0/G1 phases.

**Figure 4:**
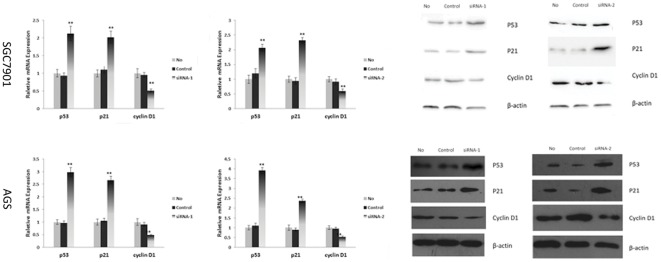
The cell cycle regulators were evaluated with Real time PCR and Western blot. Inhibition of AP-4 expression could up-regulate the expression of p53 and p21 mRNA and protein, but down-regulate the cyclin D1. (* p<0.05; ** p<0.01).

The AP-4 specific siRNAs induced cell cycle block was further investigated by observing the effects of AP-4 specific siRNAs treatment on the relative expression of cell cycle regulators: the tumor suppressor p53, the cyclin dependent kinase inhibitor p21, and the G (1)-phase-specific cyclin D1, which were critical regulators of the cell cycle and proliferation. Forty-eight hours post-transfection, human gastric cancer cells were collected for real-time PCR and immunoblotting analysis. Transfection with AP-4 specific siRNAs could up-regulate the expression of p53 and p21 protein. The modulatory effect of AP-4 siRNAs was greater than that of control siRNA (Figure 4) (p<0.01). As expected, cyclin D1 was down-regulated compared to control siRNA group and mock group (Figure 4) (p<0.01). These data further supported the hypothesis that silencing the expression of AP-4 altered the expression of other cell cycling regulators, induced cell cycle arrest and inhibited the proliferation of human gastric cancer cells

### Detection of apoptosis and the factors involved in the apoptosis pathway

**Figure 5:**
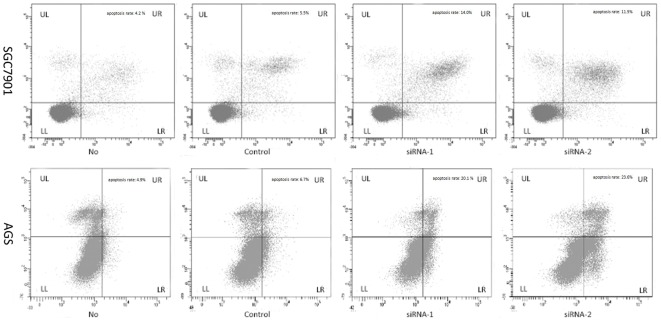
Effect of AP-4 specific-siRNAs on the induction of apoptosis in gastric cancer cells. Forty-eight hours post-transfection, the cells were harvested and double stained with Annexin-V and PI. The apoptosis rate of cells transfected with AP-4 siRNAs was higher than the control siRNA (p<0.05) and mock cells (p<0.05). (* p<0.05; ** p<0.01).

To quantify the effect of AP-4 specific siRNAs on apoptosis in human gastric cancer cells, Annexin-V and PI staining assays were used in conjunction with Flow cytometry. Cells were stained with Annexin V-FITC/PI and gated into Lower Right (LR) and Upper Right (UR) quadrants. Cells in LR and UR were considered to be early apoptotic (Annexin^+^/PI^−^) and late apoptotic (Annexin^+^/PI^+^) respectively. Cells in LL (Lower Left) and UL (Upper Left) quadrants were considered to be alive and necrotic respectively. Extent of apoptosis was expressed as the sum total of the percentages in LR and UR quadrants. The apoptotic rates were showed in Figure 5. Treated cells with AP-4 siRNAs showed more apoptotic cells (SGC7901/siRNA-1: 14.0%; SGC7901/siRNA-2:11.5%; AGS/siRNA-1: 20.1%; AGS/siRNA-2: 23.6%) than the negative (SGC7901/control siRNA: 5.5%; AGS/control siRNA: 6.7%) (p<0.05) and blank (SGC7901: 4.2%; AGS: 4.9%) (p<0.05). These results showed that AP-4 specific siRNAs were able to induce apoptosis in these cells.

**Figure 6:**
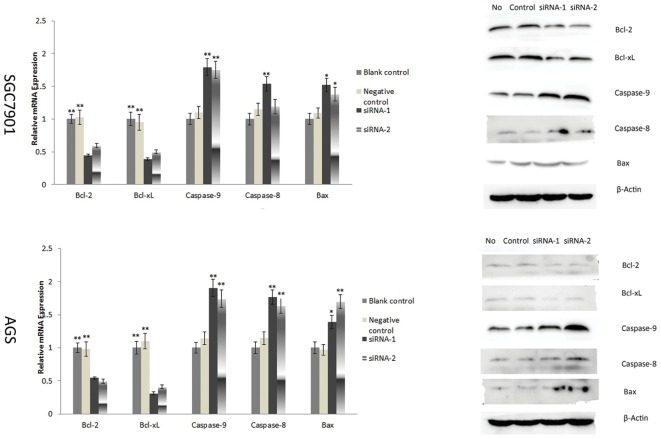
The expressions of the factors involved in the apoptosis were examined with real time PCR and Western blot. Inhibition of AP-4 expression could up-regulate the transcription of Caspase-9 but down-regulate the Bcl-2 and Bcl-x_L_ expression in human gastric cancer But Caspase-8 and Bax expression were difference in different cell lines after transfection. Forty eight hours after transfection, Caspase-8 and Bax were over expression in AGS cells. In SGC7901 cells, Bax was over expression, Caspase-8 expression was also up-regulated in siRNA-1 group, but in siRNA-2 group, Caspase-8 expression was not significant affected. (* p<0.05; ** p<0.01).

Additionally, we examined the expression of factors involved in apoptosis pathway with real-time PCR in mRNA level, such as Bcl-2, Bcl-x_L_, Caspase-9, Caspase-8 and Bax. It showed that knockdown AP-4 could up-regulate the expression of Baspase-9 in human gastric cancer cells, and the modulatory effect of AP-4 siRNAs was greater than that of control siRNA (Figure 6) (p<0.01). The expression of Bcl-2 and Bcl-x_L_ were decreased compared to the control siRNA or mock group (Figure 6) (p<0.01). These data further supported that the silencing of AP-4 expression led to apoptosis in human gastric cancer cells. However, the expression of Caspase-8 and Bax were different in different cell lines after transfection. Forty eight hours after transfection, Caspase-8 and Bax were over expression in AGS cells. In SGC7901 cells, the expression of Bax increased, but the expression of Caspase-8 increased only in siRNA-1 group. In siRNA-2 group, Caspase-8 expression was not significant affected (Figure 6).

### AP-4 silencing could regulate cell cycle and cell apoptosis in both p53-dependent and independent-manners

**Figure 7:**
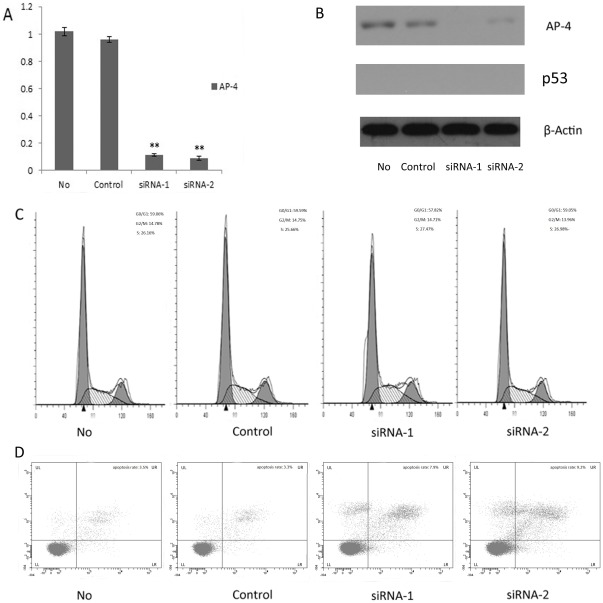
Silencing AP-4 regulated cell cycle and apoptosis in both p53-dependent and independent-manners. In Kato-III cell, the AP-4 expression was obviously down-regulated at 48 hours post-transfection. However, the induction of cell cycle arrest and apoptosis were not observed in Kato-III cells. The proportions of cells at G0/G1, G2/M and S phases were not significant difference between AP-4 siRNAs groups and control or mock groups. The apoptosis rate of Kato-III cells transfected with AP-4 siRNAs was higher than the control siRNA (p<0.05) and mock cells (p<0.05). However, the apoptosis rate in Kato-III cells with AP-4 silencing was lower than AGS and SGC7901 cells with AP-4 silencing (p<0.05).

To verify whether the up-regulation of p53 in AP-4 knockdown gastric cancer cells was critical to the role of cell cycle arrest, Kato-III cells, a kind of p53 defect gastric cancer cell line was used to evaluate the dependence of AP-4 knockdown effect on p53. We found that knockdown of AP-4 in AGS with wild-type p53 or SGC7901 with mutant p53 could induce cell cycle arrest, but this phenomenon was not observed in Kato-III cells (Figure 7), the percentage of cells at G0/G1 phases were 57.82% (siRNA-1), 59.05% (siRNA-2), 59.59% (control siRNA) and 59.06% (mock). Those indicated that knockdown AP-4 maybe regulate cell cycle by means of p53-p21 pathway. Apoptosis population was also detected in Kato-III cells with Annexin V-FITC and PI staining. The results showed that the apoptosis rate of Kato-III cells transfected with AP-4 siRNAs(siRNA-1: 7.9%; siRNA-2: 9.2%) was higher than the control siRNA(3.3%) (p<0.05) and mock cells (3.5%) (p<0.05), but to a lesser extent than it did on the AGS (p<0.05) and SGC7901 cells (p<0.05), indicating that silencing AP-4 could induce apoptosis in gastric cancer cells through both p53-dependent and independent-manners.

## Discussion

Gene expression is a fundamental and highly conserved process. Transcription, the first step in gene expression, is performed by structurally conserved DNA dependent RNA polymerases, which results in the synthesis of an RNA molecule from a DNA template [31]. Transcription factors, form transcription initiation complex with RNA polymeras II, participate in the process of transcription initiation to regulate gene expression. They may play an important role in transformation, tumorigenesis, tumor progression and metastasis by regulating transcription and therefore gene expression [32], [33], [34]. Transcription factor AP-4, belonging to the rapidly growing group of HLH proteins [8] is involved in differentiation and cellular proliferation [35], [36], [37], [38], [39], affects cell cycle events and apoptosis, regulates and controls some gene expression [9], [10], [15], [16], [17], [18], [19], [20], [21]. Recently, it has been reported that AP-4 was up-regulated in colon carcinoma, breast cancer and prostate cancer [9], [18], [24]. In addition, AP-4 positive expression indicated a poor prognosis with significance over grade, node status or size in ER+ breast cancer, and a possible association with chemo-sensitivity [40]. In our previously study, we have found that the expression of AP-4 was overexpressed and it co-related with a poor prognosis [30]. It may be a molecular marker for diagnosis and prognosis of gastric cancer. In the present study,. We designed two AP-4 specific siRNAs to inhibit the expression of the AP-4 gene in human gastric cancer cells. They were well established and transfected into cells to result in knock-down of AP-4 gene expression. We found that the most potent time point in suppressing the AP-4 expression in gastric cancer cells were 48 h and 72 h after transfection. Thus, we chose the former to further carry out related examination.

Previously, it has been shown that transcription factor AP-4, unlike other HLH proteins, contained two distinct leucine repeat elements which also direct dimerization and allow the selective complex formation of ubiquitous AP-4 protein [8], [41].

AP-4 could play an important role in modulation of cellular functions via regulation of genes involved in viral production [7], [16], [22], [42], [43], cell growth and survival [9], [17], [23], [44], immune response [14], [41], [45], and angiogenesis [21]. Peter Jung, et al found that AP-4 could encode a c-MYC-inducible repressor to inhibit p21 expression [9], and presumably played an important role in mediating the proliferative activity of c-MYC [10]. In addition, AP-4 influenced the sensitivity to apoptosis by regulating the expression of Caspase-9 [17]. In this study, we found that down-regulation of AP-4 inhibited the proliferation of human gastric cancer cells in vitro. The proliferation inhibition ratio of AP-4 specific siRNAs was significantly lower than that of the cells transfected with control siRNA or mock group.

Chemotherapy is one important strategy in the treatment of gastric cancer, but it often fails because of the resistance to anticancer drugs. It was reported that AP-4 positive expression was possibly connected with chemo-sensitivity [40]. We next investigated the role of AP-4 in the regulation of chemo-sensitivity of human gastric cancer cells and found that inhibition of AP-4 could significantly enhance the sensitivity of these cells to ADR, 5-FU or cis-plantinum treatment, suggesting that inhibition of AP-4 may have a beneficial effect in chemo-sensitivity.

In addition, silencing of AP-4, induced cell cycle arrest at G0/G1 phases, analysis of a potential mechanisms underlying the effects of the AP-4 silencing on inhibition of human gastric cancer cell proliferation were characterized by the expression of cell cycle-related regulators. We found that down-regulation of AP-4 expression inhibited the expression of cyclin D1, but up-regulated the expression of p53 and p21. p53 and p21 has been hypothesized to be a negative regulator of the cell cycle and proliferation [46], [47], on the other hand, cyclin D1 promoted progression through the G_1_-S phase of the cell cycle [48]. Down-regulated expression of AP-4, p21 and p53 in the meanwhile could make the cell cycle arrest caused knockdown of AP-4 disappearance. These results are in agreement with a model in which AP-4 induces cell cycle arrest by regulating the expression of cell cycle regulators, such as p53, p21 and cyclin D1.

Apoptosis, or programmed cell death, is known to participate in various biological processes by two main apoptotic pathways, the mitochondrial (intrinsic) pathway and the death receptor (extrinsic) pathway [49]. We found that silencing the AP-4 expression trigged cell apoptosis in our experiment, which demonstrated that AP-4 suppressed apoptosis in human gastric cancer cells.

In our experiment, increasing levels of Caspase-9 and down-regulation of Bcl-2 and Bcl-x_L_ were detected in human gastric cancer cells, indicating that knockdown of AP-4 activated both intrinsic and extrinsic pathways to apoptosis in cancer cells. [49], [50]

In summary, the data demonstrate that RNAi-mediated down-regulation of transcription factor AP-4 effectively inhibited the cell proliferation, indicated cell cycle arrest, triggered apoptosis and enhanced chemo-sensitivity of human gastric cancer cells with the decreased expression of cyclin D1, Bcl-2 and Bcl-x_L_ and activated p21, p53 and Caspase-9 expression, which suggested AP-4 may be a oncogene playing an important role in tumorigenesis. Although the precise mechanism of this role needs to be further investigated, the AP-4specific-siRNAs may be of potential values as novel therapeutic agents for human gastric cancer.

## Materials and Methods

### Cell line and cell culture

Human gastric cancer cell lines AGS with wild-type p53, SGC7901 with mutant p53 (obtained from Wuhan University) and Kato-III with p53 genome deletion (Zhiyan Bio Technology Co, Shanghai) were cultured in DMEM medium or RPMI1640 medium (Invitrogen) containing with 10% fetal bovine serum (Invitrogen), penicillin (100 U/ml) and streptomycin (100 µg/ml). Cells were maintained at 37°C in a humidified atmosphere of 5% CO_2_.

### Specific siRNA and transfection

The cDNA sequence of the AP-4 gene was obtained from Genbank (NM_003223) and the targeting sequences of two 21-nucleotide different siRNAs were designed and chemically synthesized (Qiagen Germany). The nucleotide sequences were as follows: siRNA-1, 5′-CGGGAUUCCAGUCCCUCAATT-3′ (sense), and 5′-UUGAGGGACUGGAAUCCCGCG-3′ (antisense). siRNA-2, 5′-UGGGAUUGUCAGCCUUCAATT-3′ (sense), and 5′-UUGAAGGCUGACAAUCCCAGG-3′ (antisense). Allstars negative control siRNA (Qiagen Germany) were used as a scrambled siRNA control. Cells were plated in 6-well plates and the siRNAs were transfected into culture cells with Lipofectamine 2000 (Invitrogen) according to the manufacturer's instructions.

### Real-time quantitative PCR

Total RNA extraction was performed using RNAiso Plus (Takara, Japan) according to the manufacturer's protocol. The cDNAs from total RNA were synthesized using PrimeScript® RT reagent Kit (Takara, Japan). The mRNA expression was evaluated by real-time PCR on an ABI StepOne Plus (Applied Biosystems, Singapore) with Fast SYBR Green PCR reagents. GAPDH was applied as the internal control. The concentrations of the reagents were adjusted to reach a final volume of 20 µL, containing 2 µL reverse-transcribed product, 10 µl of Fast SYBR® Green Master Mix (Applied Biosystems, Foster City, CA), and 0.5 µl of 10 µM forward and reverse primers. The reaction was carried out by 45 amplification cycles of 95°C for 3 s and 60°C for 30 s. The following primers were designed (Table.1). PCR primers were designed by Primer 5.0 and Blast search to check specificity. Primer sequences used are listed in Tables 1. The results were calculated by using 2^−ΔΔCt^ method.

**Table 1:** Primer series of AP-4, p21, p53, Cyclin D1, Bcl-2, Bcl-x_L_, Caspase-9, Caspase-8, Bax and GAPDH gene.

Gene	Forward sequence	Reverse sequence
**AP-4**	GAGCCAGCCTGGGATTGTC	GTGCTTAAAGGAGAAAGAAGAAAACC
**p21**	AGGTGGACCTGGAGACTCTCAG	TCCTCTTGGAGAAGATCAGCCG
**p53**	CCTCAGCATCTTATCCGAGTGG	TGGATGGTGGTACAGTCAGAGC
**Cyclin D1**	TCTACACCGACAACTCCATCCG	TCTGGCATTTTGGAGAGGAAGTG
**Bcl-2**	ATCGCCCTGTGGATGACTGAGT	GCCAGGAGAAATCAAACAGAGGC
**Bcl-x_L_**	GCCACTTACCTGAATGACCACC	AACCAGCGGTTGAAGCGTTCCT
**Caspase -9**	GTTTGAGGACCTTCGACCAGCT	CAACGTACCAGGAGCCACTCTT
**Caspase -8**	AGAAGAGGGTCATCCTGGGAGA	TCAGGACTTCCTTCAAGGCTGC
**Bax**	TCAGGATGCGTCCACCAAGAAG	TGTGTCCACGGCGGCAATCATC
**GAPDH**	TGTTGCCATCAATGACCCCTT	CTCCACGACGTACTCAGCG

### Western blot

Protein was extracted with protein extraction kit (Beyotime, China) according to the direction. The protein was diluted to 3 µg/µl and was separated by 10% SDS-PAGE, transferred to polyvinylidene difluoride (PVDF) membranes (Millipore, USA), and then was blocked in 5% non-fat powdered milk in TBST for 1 hour at room temperature. Immunoblotting was performed using anti-AP-4 antibody (Sigma-aldrich, Shanghai, dilution 1∶1000), anti-p21 antibody (Sigma-aldrich, Shanghai, dilution 1∶1000), anti-p53 antibody (Abcam, UK, dilution 1∶500) and anti-cyclin D1 antibody (Abcam, UK, dilution 1∶500) overnight at 4°C. After three times rinsed with TBST, the membrane was incubated with horseradish peroxidase-conjugated secondary antibodies (dilution 1∶2000, Boster, China) for 1 hour at room temperature. The outcome was visualized by the ECL Plus Western blotting detection system according to the manufacturer's instructions. Anti-β-actin (dilution 1∶1000, Boster, China) antibody acted as internal control.

### Measurement of cell proliferation

The impact of silencing AP-4 on the gastric cancer cell proliferation after 48 hours of transfection was measured by Cell Counting Kit-8(CCK-8) (Beyotime, China), according to the manufacturer's instruction. Briefly, gastric cancer cells were cultured in 96-well plates and transfected with 20 nM of control siRNA, AP-4 specific siRNAs. After 48 hours, 10 µl of CCK-8 reagent was added to each well, After 1 hour of incubation at 37°C, the absorbance measured at 450 nm. The relative levels of cell proliferation in each group of cells was calculated according to the following formula: R = (A2-A1)/A2×100% and P = A1/A2×100% in which R was relatively inhibitory rate and P was relatively proliferation ratio of cell growth; A1was mean absorbance value of transfected cells; and A2 was mean absorbance value of untransfected control cells without any drug treatment. All experiments were done with 5 wells per experiment and repeated at least three times.

### Cell cycle Assay

Cells were harvested 48 hours after transfection and fixed in 70% ice-cold ethanol overnight, washed with 1×PBS, and stained with propidium iodide (PI) (50 µg/ml) in 1×PBS supplemented with RNase (50 µg/ml) for 30 minutes. Tests were performed in triplicate for each sample, and analyses were performed by flow cytometer (FACS CantoII, BD Bioscience, USA) in accordance with the manufacturer's guidelines.

### Chemo-sensitivity test in vitro

As described previously, CCK-8 assay was used to assess effect of the chemo-sensitivity of gastric cancer cells to anticancer drugs. In brief, six hours after transfection, the medium was removed and replaced with fresh medium containing varying concentrations of anti-tumor drug (ADR, 5-FU or cis-platinum) and incubated for 48 hours. Relatively inhibitory rate of cell growth was calculated according to the formula listed above.

### Apoptosis assay by Annexin V-FITC and propidium iodide (PI) staining

To assess the rate of cell apoptosis, apoptosis was quantified by annexin-V–FITC and propidium iodide double staining using an Annexin-V/FITC kit (Antgene, China). Cells were collected according to the manufacturer's instructions 48 hours after transfection, washed with cold PBS, and suspended in binding buffer, and then the cells were incubated 30 minutes in the dark at 4°C with Annexin V-FITC and PI in phosphate buffer and analyzed on the flow cytometer (FACS CantoII, BD Bioscience, USA) within 1 h after staining.

### Statistical Analysis

All data were shown as mean ± SD. Difference among groups was analyzed by one-way ANOVA and Student–Newman–Keuls (SNK)-q test using a SPSS 12.0 for Windows software. Statistical significance was defined as * p<0.05 and ** p<0.01.
